# Supplementary material for: Empirical methods for controlling false positives and estimating confidence in ChIP-Seq peaks
Source: BMC Bioinformatics. 2008 Dec 5;9:523. doi: 10.1186/1471-2105-9-523 (PMC2628906; doi:10.1186/1471-2105-9-523)
Supplement: Additional File 1 — A variety of html documents from the USeq web site detailing the available applications, their best usage, output file type descriptions, command line menus, etc. [file 1471-2105-9-523-S1.zip › USeqUserGuides/JavaDoc.zip!/JavaDoc/trans/cel/MakeChromosomeSets.html]

MakeChromosomeSets


|  |  |  |
| --- | --- | --- |
| **Overview** \| **Package** \| **Class** \| **Use** \| **Tree** \| **Deprecated** \| **Index** \| **Help** | |  |
| **PREV CLASS**   **NEXT CLASS** | **FRAMES**    **NO FRAMES**     **All Classes** |  |
| SUMMARY: NESTED \| FIELD \| CONSTR \| METHOD | DETAIL: FIELD \| CONSTR \| METHOD |  |


---


## trans.cel Class MakeChromosomeSets

```
java.lang.Object
  trans.cel.MakeChromosomeSets
```

---

``` public class MakeChromosomeSets extends java.lang.Object ```

---

**Constructor Summary**

`MakeChromosomeSets(java.lang.String[] args)`


| **Method Summary** | |
| --- | --- |
| `static IntensityFeature[]` | `collapseIntensityFeatureArray(IntensityFeature[][] fs)`             Collapses an uneven array of IntensityFeature[][] to IntensityFeature[]. |
| `static java.io.File[]` | `fetchChromosomeFiles(java.io.File[] chipDir, java.lang.String chromosomeName)`             Extracts all the Files with a given chromosome name from a list of directories |
| `static IntensityFeature[]` | `fetchIntensityFeatures(java.io.File[] f)`             Loads IntensityFeature[]s into a single master. |
| `static void` | `main(java.lang.String[] args)` |
| `long` | `makeSets(java.io.File[] chipSetDirectories, java.io.File saveDirectory)`             Merges chromosome info together and saves as whole chromosomes, will also save the oligoPositions if oligoPositionsDirectory isn't null. |
| `static void` | `printDocs()` |
| `void` | `processArgs(java.lang.String[] args)`             This method will process each argument and assign any new varibles |
| `static void` | `splitAndSaveIntensities(IntensityFeature[] ints, java.io.File f)`             Saves the intensities to file as a int[] |
| `static void` | `splitAndSavePositions(IntensityFeature[] ints, java.io.File f)`             Saves the positions to file as a int[] |

**Methods inherited from class java.lang.Object**

`equals, getClass, hashCode, notify, notifyAll, toString, wait, wait, wait`

**Constructor Detail**

### MakeChromosomeSets

```
public MakeChromosomeSets(java.lang.String[] args)
```


**Method Detail**

### makeSets

```
public long makeSets(java.io.File[] chipSetDirectories,
                     java.io.File saveDirectory)
```

:   Merges chromosome info together and saves as whole chromosomes, will also save the oligoPositions if oligoPositionsDirectory isn't null.
    Returns the total number of oligos found across all chromosomes.

---


### splitAndSaveIntensities

```
public static void splitAndSaveIntensities(IntensityFeature[] ints,
                                           java.io.File f)
```

:   Saves the intensities to file as a int[]

---


### splitAndSavePositions

```
public static void splitAndSavePositions(IntensityFeature[] ints,
                                         java.io.File f)
```

:   Saves the positions to file as a int[]

---


### fetchIntensityFeatures

```
public static IntensityFeature[] fetchIntensityFeatures(java.io.File[] f)
```

:   Loads IntensityFeature[]s into a single master.

---


### collapseIntensityFeatureArray

```
public static IntensityFeature[] collapseIntensityFeatureArray(IntensityFeature[][] fs)
```

:   Collapses an uneven array of IntensityFeature[][] to IntensityFeature[].

---


### fetchChromosomeFiles

```
public static java.io.File[] fetchChromosomeFiles(java.io.File[] chipDir,
                                                  java.lang.String chromosomeName)
```

:   Extracts all the Files with a given chromosome name from a list of directories

---


### processArgs

```
public void processArgs(java.lang.String[] args)
```

:   This method will process each argument and assign any new varibles

---


### printDocs

```
public static void printDocs()
```

---


### main

```
public static void main(java.lang.String[] args)
```


---


|  |  |  |
| --- | --- | --- |
| **Overview** \| **Package** \| **Class** \| **Use** \| **Tree** \| **Deprecated** \| **Index** \| **Help** | |  |
| **PREV CLASS**   **NEXT CLASS** | **FRAMES**    **NO FRAMES**     **All Classes** |  |
| SUMMARY: NESTED \| FIELD \| CONSTR \| METHOD | DETAIL: FIELD \| CONSTR \| METHOD |  |


---
